# Brain Atrophy and Hypomyelination Associated with Iatrogenic Cushing Syndrome in an Infant 

**Published:** 2018

**Authors:** Sumeyra DOGAN, Mehmet S DOGAN, Filiz TUTUNCULER, Ozge YAPICIUGURLAR, Hakan GENCHELLAC

**Affiliations:** 1 Department of Pediatric Radiology, Trakya University, Faculty of Medicine, Edirne, Turkey.; 2 Department of Pediatric Endocrinology, Faculty of Medicine, Trakya University, Edirne, Turkey.

**Keywords:** Diaper dermatitis, Iatrogenic Cushing’s syndrome, Brain atrophy, Myelination, Magnetic resonance imaging

## Abstract

Prolonged use of topical corticosteroids, particularly in infants, albeit rare, may lead to Cushing syndrome. Central nervous system abnormalities including brain atrophy and delayed myelination on cranial magnetic resonance imaging has been reported in patients with corticosteroid treatment. We herein report a 5-month-old female infant referred to Department of Pediatric Endocrinology, Edirne, Turkey with brain atrophy and myelination delay that might be due to iatrogenic Cushing syndrome caused by topical corticosteroid use.

## Introduction

Cushing syndrome (CS) is a rarely seen entity in childhood. Iatrogenic or exogenous CS due to chronic administration of glucocorticoids is the most common form of CS in children. Peroral or parenteral forms of glucocorticoids are more likely to cause CS than topical glucocorticoids (1). However, infants are more vulnerable to the side effects of topical glucocorticoids compared to older individuals because they have higher ratio of body surface area to body weight, and thinner skin barrier (2, 3). Therefore, long term use of potent topical corticosteroids may lead to iatrogenic CS in infants (2-7). 

Brain atrophy has been reported in both pediatric and adult patients being treated with steroids with the indication of respiratory, rheumatological, and autoimmune diseases (8). Few cases have been reported regarding CS caused by topical steroid misuse (2-7). However, to our knowledge, associated imaging findings of brain atrophy and myelination delay have not been reported in children. 

We herein report a 5-month-old infant with brain atrophy and myelination delay associated with iatrogenic CS caused by topical corticosteroid use.

## Case Report

**Fig.1:**
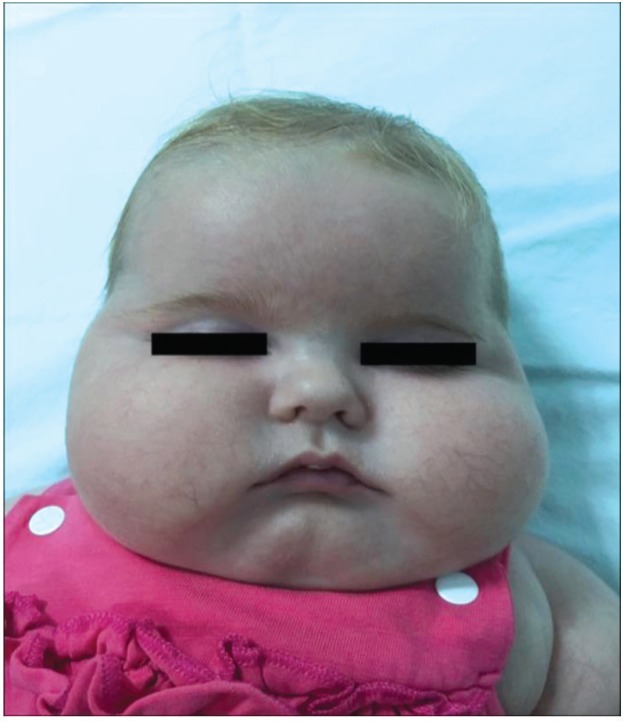
Typical cushingoid face of the patient

A 5-month-old girl was referred to Department of Pediatric Endocrinology, Edirne, Turkey with the complaint of excessive weight gain (1300 g in one month). She was born at term as the first child of the family. Her birth and family history were otherwise unremarkable. Her birth weight, height, and head circumference were within normal limits. On physical examination her weight was 6.9 kg (50-75^th ^percentile), length was 56.8 cm (3^rd^-10^th^ percentile), and head circumference was 38.5 cm (3^rd^ percentile). While body temperature was within normal limits, blood pressure was above the 95^th^ percentile for this age group (135/102 mmHg). Tachycardia and tachypnea were also present. She had cushingoid face, buffalo hump, truncal obesity, paperthin skin, and telengiectasies involving her face and body (Fig. 1).

Detailed history obtained from her mother revealed unprescribed usage of a topical steroid (clobetasol-17-propionate) for diaper dermatitis for last two months in every diaper change with the recommendation of her grandmother. There was no history of any oral or parenteral medication. Laboratory data revealed trombocytosis (618000/mm^3^) and elevated levels of aspartate aminotransferase (104 UI/L). Basal serum cortisol and adrenocorticotropic hormone (ACTH) levels were low (0.8 µg/dL and 8.6 pg/mL, respectively) consistent with iatrogenic Cushing sydrome. To exclude tertiary adrenal insufficiency, low dose ACTH stimulation was performed. As 1 µg ACTH stimulation test was normal with maximum cortisol of 19.4 µg/dL, tertiary adrenal insufficiency was excluded and hydrocortisone replacement therapy was not started. Other blood test results and urinalysis were within normal limits. Abdominal ultrasonography was normal except for grade 1 hepatosteatosis. On the seventh day of hospitalization she was presented with fever, fontanelle bulging, lethargy, feeding problems, and vomitting. 

**Fig.2.a-c.**
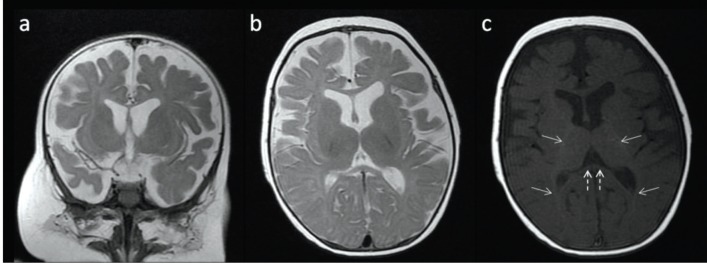
On coronal (a) and axial (b) T2W images, prominent intra- and extraaxial subarachnoid spaces and decreased cerebral volume consistent with brain atrophy are seen. Hyperintensity on T1W images resembling myelination is seen at posterior limbs of internal capsules and optic radiations (arrows). There are no evidence of T1 hyperintensities at the splenium of corpus callosum which is expected to be myelinated by the age of 4 months (dashed arrows) (c). Note the thickened subcutaneous fat in all series

On cranial magnetic resonance imaging (MRI) obtained to rule out the increased intracranial pressure, prominent intra- and extraaxial subarachnoid spaces and decreased cerebral volume consistent with brain atrophy were detected. Furthermore, delayed myelination was also encountered (Fig. 2). Leukocytosis was detected (11800/mm^3^) on complete blood count. Although the lumbar puncture revealed a normal level of white blood cell count, lower cerebrospinal-to-serum glucose ratio was also noted. Thus, acute bacterial meningitis could not be excluded and intravenous ceftriaxone (100 mg/kg/day) was started. Her symptoms resolved gradually. Antibiotherapy was continued for ten days although cerebrospinal fluid culture was negative. Subsequently, she was discharged with the advice of 1 month follow-up. In the follow up controls, the Cushingoid appearance recovered completely at 9-month-old. When she reached one-year-old, she could stand on her own and started to walk, she could use spoon, she was able to hold a pen and scribble, understand and say some words. Therefore she was considered at the same level as her peers in terms of neuromotor development. The follow-up controls of the patient in healthy child polyclinic are ongoing. 

Informed consent was taken from her parents and the study was approved by Ethics Committee of the hospital.

## Discussion

In current case, misuse of a potent topical corticosteroid for diaper dermatitis may be responsible for CS, brain atrophy, and delayed myelination. Iatrogenic CS caused by topical corticosteroid use is a rare entity that has been reported mostly in infants with diaper dermatitis (2, 7). The long term administration of topical corticosteroids with high potency to the skin covered with diaper or clothes are the risk factors that may cause systemic side effects like CS and adrenal suppression (7). Our patient had many of these risk factors. 

One of the most potent topical steriod, clobetasol-17-propionate was applied after every diaper change for two months. In a study recruiting 18 children who had CS due to using nappy rash ointments, clobetasol was the most frequently used agent with the percentage of 73%. In this study, the duration of application of steroid including ointments was variable, with the period of at least 3 weeks in one patient to more than 1 year in 3 patients. Another result of this study was the high rate (72% of the cases) of self medication (7). Self medication is a common public health problem in developing countries that may lead to severe health problems as discussed in this case. If necessary, these products should be used under the supervision of physicians and parents should be informed about the use and potential side effects of the drug. Furthermore, purchasing of these products from the drugstores without prescription should be prohibited.

There are also other systemic side effects of corticosterids apart from CS including hypertension, dyslipidaemia, adrenal insufficiency, failure to thrive, skin atrophy, striae, cataract, glaucoma, and predisposition to infections (3-6). At presentation, our patient had hypertension and findings indicating failure to thrive. Adrenal insufficiency was excluded in the light of normal low dose ACTH stimulation test. Furthermore, long-term application of the topical corticosteroid might have lead to tendency to infection that was considered as meningitis in our case. Our search for literature revealed a case of urinary infection and two cases of disseminated cytomegalovirus infection due to misuse of topical corticosteroid in children (4-6).

MRI abnormalities including brain atrophy and delayed myelination have been reported in both pediatric and adult patients being treated with corticosteroids (8). The evidence of dose-dependent brain atrophy and myelination delay was also shown in animal studies that investigated the side effects of antenatal administration of corticosteroids (9). The pathogenetic mechanisms of brain atrophy and myelination delay caused by chronic corticosteroid treatment is not well-known (8, 9). Glucocorticoids are thought to play inhibitory role in the neuronal maturation and myelination process by restraining the differentiation of oligodendrocyte precursors (9). Reduced glucose uptake in the brain, increased effects of excitatory amino acid neurotransmitters, decline in neurotrophic factors, and decrease in neurogenesis are deemed as mechanisms that account for brain atrophy (8). MRI is not only a very useful tool to show the details of brain anatomy without exposure to radiation, but also the best diagnostic modality to evaluate myelination of the developing brain (10). T1 is the most valuable sequence for demonstrating myelination in the first year of life, whereas T2-weighted (T2W) images provide additional information in later stages of myelin maturation. Myelinated white matter is seen hyperintense on T1-weighted (T1W) images consistent with increasing amount of cholesterol and galactocerebroside within myelin membranes, and hypointense on T2W images owing to reduced free water content between hydrophobic myelin sheats in maturing white matter (10). In our patient, normal myelination was only seen at bilateral perirolandic centrum semiovale, posterior limbs of internal capsules, optic radiations, brainstem, and deep cerebellar white matter on T1W images. Although the splenium of corpus callosum is expected to be myelinated and seen hyperintense on T1W images by the age of 4 months (10), there was no evidence of T1 hyperintensity at these areas in this 5-month-old infant. Therefore delayed myelination was considered in addition to brain atrophy.

**In conclusion**, this is the unique case of iatrogenic CS with the findings of brain atrophy and delayed myelination demonstrated on MRI. The physicians should be well aware of potential systemic and neurodevelopmental side effects of topical steroids.

## Authors’ Contribution:

Sumeyra Dogan: Drafting, designing of the work, analysis, collecting radiological data and final approval of the work.

Mehmet S Dogan: Drafting, designing of the work, analysis, and final approval of the work.

Filiz Tutunculer: Clinical evaluation of the patient, Designing of the work, interpretation and final approval of the work.

Ozge Yapici Ugurlar: Designing of the work, collecting radiological data, analysis, and final approval of the work.

Hakan Genchellac: Designing of the work, interpretation and final approval of the work.

All authors agreed to be accountable for all aspects of the work in ensuring that questions related to the accuracy or integrity of any part of the work are appropriately investigated and resolved.
